# Response of Extreme Rainfall for Landfalling Tropical Cyclones Undergoing Extratropical Transition to Projected Climate Change: Hurricane Irene (2011)

**DOI:** 10.1029/2019EF001360

**Published:** 2020-03-03

**Authors:** M. Liu, L. Yang, J. A. Smith, G. A. Vecchi

**Affiliations:** ^1^ Department of Civil and Environmental Engineering Princeton University Princeton NJ USA; ^2^ School of Geography and Ocean Sciences Nanjing University Nanjing China; ^3^ Department of Geosciences Princeton University Princeton NJ USA; ^4^ Princeton Environmental Institute Princeton University Princeton NJ USA

**Keywords:** tropical cyclone rainfall, climate change, extratropical transition

## Abstract

Extreme rainfall and flooding associated with landfalling tropical cyclones (TCs) have large societal impacts, both in fatalities and economic losses. This study examines the response of TC rainfall to climate change projected under future anthropogenic greenhouse emissions, focusing on Hurricane Irene, which produced severe flooding across the Northeastern United States in August 2011. Numerical simulations are made with the Weather Research and Forecasting model, placing Irene in the present‐day climate and one projected for the end of 21st century climate represented by Phase 5 of the Coupled Model Intercomparison Project Representative Concentration Pathway 8.5 scenario. Projected future changes to surface and atmospheric temperature lead to a storm rainfall increase of 32% relative to the control run, exceeding the rate expected by the Clausius‐Clapeyron relation given a ~3‐K lower atmospheric warming. Analyses of the atmospheric water balance highlight contributions to the increase in rainfall rate from both increased circulation strength and atmospheric moisture. Storm rainfall rate shows contrasting response to global warming during TC and extratropical transition periods. During the TC phase, Irene shows a significant increase of storm rainfall rate in inner core regions. This increase shifts to outer rainbands as Irene undergoes extratropical transition, collocated with the maximum tangential wind increase and the change of secondary circulation strength. Changes of storm track from the control run to global warming projections play a role in the change of spatial rainfall pattern. Distinct roles of surface and atmospheric warming in storm rainfall and structure changes are also examined.

## Key Points

- Increase of Irene rain rate is observed in inner core regions for tropical cyclone phase but in outer rainbands during transition period
- Increase of Irene rain rate exceeds the rate of water vapor increase in a warming climate, suggesting the importance of dynamical effects

## 1 Introduction

Tropical cyclones (TCs) are among the most devastating natural disasters in terms of both fatalities and economic impacts (e.g., Chavas et al., 2013; Rappaport, 2000; Rappaport, 2014). Improved understanding of drivers for changes of TC activity is critical to advance the projection of future TC hazards. These changes will involve, among other factors, the response of TCs to climate changes driven by radiative forcing changes from anthropogenic emissions. Recent efforts to understand these responses have been undertaken using atmospheric general circulation models (Murakami et al., 2011; Murakami et al., 2012; Murakami & Wang, 2010), coupled general circulation models (Broccoli & Manabe, 1990; Camargo, 2013; Gualdi et al., 2008; Liu et al., 2018), dynamical downscaling approaches (Bender et al., 2010; Knutson et al., 2008; Knutson et al., 2013; Knutson et al., 2015), and statistical dynamical approaches (Emanuel, 2013; Emanuel et al., 2008).

An important aspect of TC‐related hazards is riverine flooding associated with extreme rainfall (e.g., Liu & Smith, 2016; Smith et al., 2005; Smith et al., 2010; Smith et al., 2011; Villarini, Goska, et al., 2014; Villarini & Smith, 2010). The change of TC rainfall rate in response to warming driven by past and future anthropogenic forcing shows increases across a range of climate models and warming scenarios (e.g., see Knutson et al., 2010, and Intergovernmental Panel on Climate Change, 2013, for a comprehensive review). With numerical experiments involving sea surface temperature (SST) increase and CO_2_ doubling in three global atmospheric models, Villarini, Lavers, et al. (2014) examined the change of storm rainfall rate across various ocean basins and found an increase of 10–20%. Scoccimarro et al. (2014) examined landfalling TCs based on the same set of model experiments as Villarini, Lavers, et al. (2014) and reported increased storm rainfall.

Dynamical downscaling approaches with regional climate models provide another important path for simulation and projection of TC activity due to the use of fine spatial resolutions. Downscaling studies using the Geophysical Fluid Dynamics Laboratory regional dynamical model found an increase of storm rainfall rate within the radius of 100 km on the order of 20% by the end of 21st century in the North Atlantic, surpassing the rate of water vapor increase expected by the Clausius‐Clapeyron relation (Knutson et al., 2008; Knutson et al., 2013). Knutson et al. (2015) extended the work by Knutson et al. (2013) to the global scale with a similar framework and found increased storm rainfall in global average but with varying rates across basins. Wright et al. (2015) adopted the model experiments introduced in Knutson et al. (2013) but focused on landfalling TCs in the eastern United States. They found increased storm rainfall over both ocean and land with the rate of change at least comparable to the expectation from the Clausius‐Clapeyron relation.

A disadvantage of many dynamical models is the use of a relatively coarse horizontal grid spacing (often limited by computational power), which accompanies convection parameterizations that do not allow explicitly resolving convective processes. Knutson and Tuleya (2004) studied the sensitivity of the responses of TC rainfall to CO_2_‐induced warming and found that the fractional change of TC rainfall is more sensitive to the convective scheme than to the fractional change of intensity, highlighting the importance of dynamical models that allow explicit convection. Hill and Lackmann (2011) carried out pseudo‐global‐warming downscaling simulations to study future climate change impacts on TC intensity and rainfall by injecting the climate change signal from Representative Concentration Pathway (RCP) 8.5 scenario into a high‐resolution numerical model. Wang et al. (2015) applied a similar methodology to two landfalling typhoons in Taiwan to examine the impact of past long‐term climate change on TC rainfall rate. These studies found that the enhanced upper tropospheric warming and SST increase play an offsetting role in storm response to climate change (Shen et al., 2000). A similar approach was also applied to Hurricane Sandy (2012) (Lackmann, 2015). It was found that thermodynamic changes do not play a critical role in the observed strength of Sandy. The substantial strengthening of Sandy under Intergovernmental Panel on Climate Change Fourth Assessment Report (AR4) A2 emissions scenario, on the other hand, highlights the role of projected global warming (GW) on TC intensity.

Extratropical transition (ET) is a key contributor to extreme rainfall and associated riverine flooding from landfalling TCs, particularly in the northeastern United States, as illustrated by Hurricane Agnes (1972) (DiMego & Bosart, 1982), Hurricane Floyd (1999) (Atallah & Bosart, 2003; Colle, 2003), and Hurricane Irene (2011) (Liu & Smith, 2016). The response of transitioning storms to anthropogenic greenhouse gas emissions has important implications for future flood risks for inland and coastal communities, which compared to other aspects of TCs has yet received less attention (Baatsen et al., 2015; Haarsma et al., 2013; Jung & Lackmann, 2019; Liu et al., 2017, 2018). Hurricane Irene (2011), which produced severe flooding, provides an ideal case for examining the response of transitioning storms to climate warming since ET is a key element of extreme rainfall from Irene (Liu & Smith, 2016). We do not aim to answer questions involving the frequency change of transitioning storms but rather to examine the influence of environmental thermodynamic changes on storm rainfall rate and associated storm structures. Jung and Lackmann (2019) conducted similar analyses to investigate the ET activity of Irene in a warmer cliamte. Unlike Jung and Lackmann (2019), we focus more on the contrasting responses of Irene rainfall and underlying physical mechanism before and during ET to climate warming. We used the cyclone phase space (CPS; Evans & Hart, 2003; Hart, 2003) method to determine the timings of ET onset and completion for Irene. The storm period before and after ET onset was treated as TC phase and ET phase, respectively. The storm period after ET completion for Weather Research and Forecasting (WRF) simulations ranges between 2 and 5 hr, which are much shorter than the entire simulation period (54 hr) and were included in ET phase for analyses. We then examined the contrasting patterns of rainfall changes for the two phases. In addition, the separate influences of surface and atmospheric warming on storm rainfall rate change of the transitioning TC were examined by designing different versions of future Irene simulations. More details of the simulation setup are discussed later.

We introduce the experimental design in section 2. Section 3 presents the results. A summary and conclusions are provided in section 4.

## 2 Methodology

### 2.1 Model Configuration

**Figure 1:**
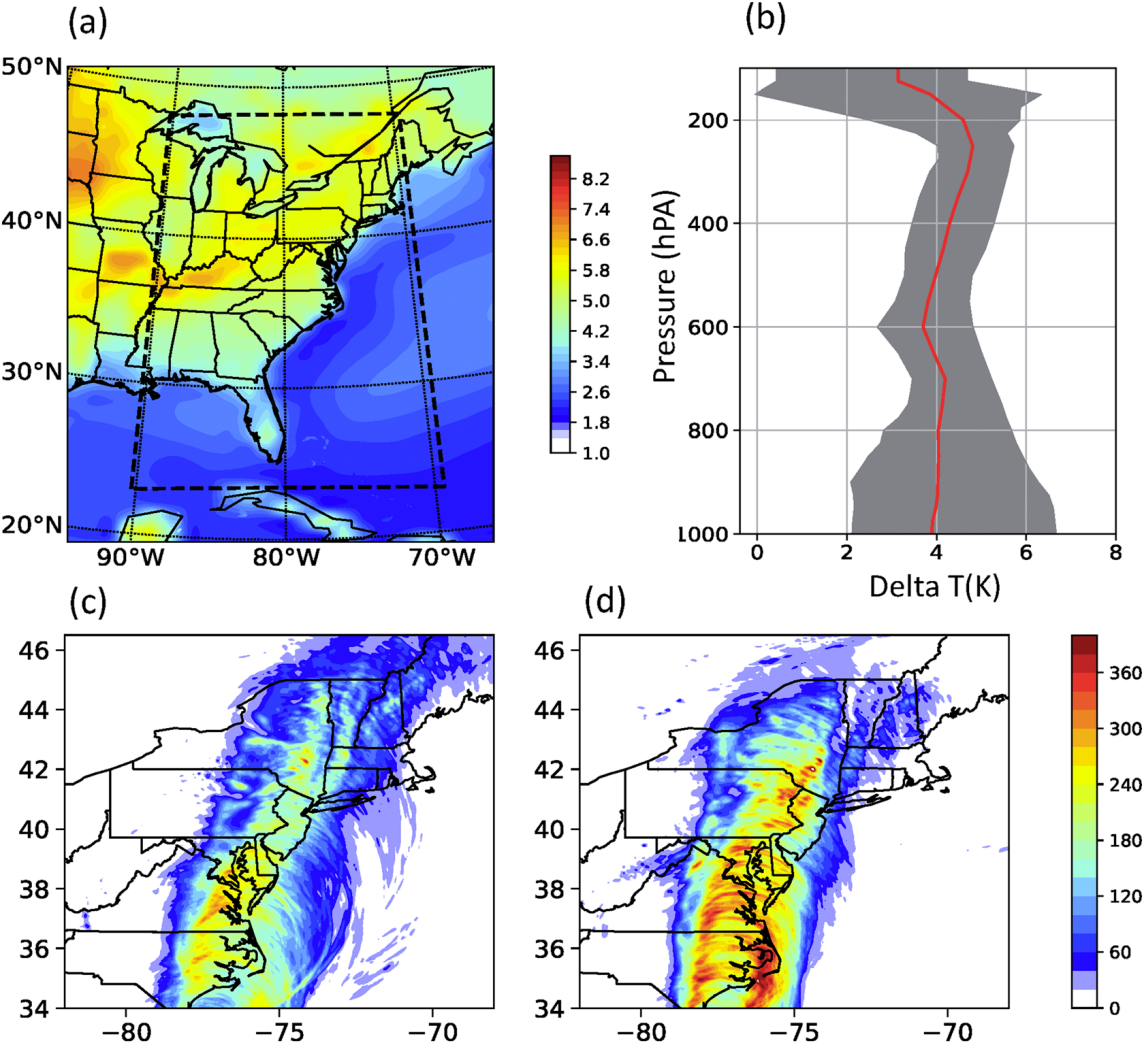
(a) The two one‐way nested WRF domains superimposed on changes of surface skin temperature (K) between global warming and control scenario. The border of the figure shows the outer domain boundary, while the dashed lines show the inner domain boundary. (b) Vertical profile of changes in air temperature (K) within the inner domain. Red line represents the mean value, while gray shading represents the one standard deviation spread. (c and d) Storm total rainfall (mm) of Hurricane Irene (2011) in the control simulation and “pseudo‐global‐warming” scenario using both surface and air temperatures, respectively.

The WRF model, developed by the National Center for Atmospheric Research, is a fully compressible, nonhydrostatic, mesoscale model. The Advanced Research version of WRF (Version 3.6.1) was used to perform numerical simulations of Hurricane Irene. We developed two one‐way nested domains (Figure 1a) with horizontal grid spacing of 9 and 3 km, respectively. Our analyses focus on the inner domain that allows explicit convection. Detailed physical options are provided in Table S1 in the supporting information.

A previous study has demonstrated the skill of WRF in reproducing many key aspects of Irene including spatial and temporal rainfall pattern and the ET process (Liu & Smith, 2016), providing the basis for examining Irene's response to future anthropogenic forcing. We first performed a control simulation to reproduce the condition in which Irene occurred by using 32‐km, 3‐hr National Centers for Environmental Prediction North American Regional Reanalysis for initial and boundary conditions (Liu & Smith, 2016). We also performed numerical experiments to simulate what would occur if Irene occurred at the end of 21st century under RCP8.5 scenario through a “pseudo‐global‐warming” approach in which the thermodynamic component of the climate change signal was added to historical input data used in the control simulation. Climate simulations from Community Earth System Model (CESM) were used to provide anthropogenic climate change by computing the difference between the 2080–2100 August climatology from CESM RCP8.5 projection and 1990–2010 August climatology from CESM historical run, which was added to historical lateral and initial conditions for the WRF model to produce the “pseudo‐global‐warming” version of Irene.

The variables adjusted for model run include surface temperature and air temperature. Changes in atmospheric water vapor content are accounted for by assuming negligible change of relative humidity (Held & Soden, 2006). Imposing thermodynamic changes may lead to imbalance between mass and wind fields. It has been found that imbalances between the wind and mass field are sufficiently small to produce spurious gravity waves in early hours of simulation (Lackmann, 2013; Marciano, Lackmann, & Robinson, 2015). A study examining the response of Hurricane Sandy to warming climate adopted the WRF digital filter initialization procedure to balance the wind and mass fields at the initial time, which led to reductions of spin‐up time (Lackmann, 2015). Comparison of simulated storm tracks from WRF runs with and without digital filter initialization showed little difference (Lackmann, 2015). In this study, we treated the first 12 hr of our simulations as model spin‐up and excluded them from analyses.

We focus on the influence of thermodynamic changes on TC rainfall, rather than aiming to make real projections of TC rainfall under warming climate. Adding future wind fields could induce perturbations of vertical wind shear that is expected to influence simulated storm intensity and rainfall. Our study represents an evaluation of future storm changes under similar shear conditions to current climate, which may become more likely under warming climate (Vecchi & Soden, 2007). On the other hand, although we imposed thermodynamic changes on the current synoptic environment, the future synoptic environment is allowed to evolve dynamically in response to thermodynamic changes, the influence of which on Irene include dynamical changes as well.

Motivated by the compensating influences of upper atmospheric warming and SST increase on future TC change, we designed three versions of Irene simulations for future scenarios. The first GW simulation employed the change of land and SST, named as GW_ST. The second simulation employed the change of air temperature throughout the troposphere to solely account for the impact from tropospheric warming, named as GW_AirT. The final simulation employed both surface and air temperatures, named as GW.

### 2.2 CPS

The CPS (Evans & Hart, 2003; Hart, 2003) method was used to determine the ET onset and completion time of Hurricane Irene. The method shows good skill in characterizing the ET process of Irene (Liu & Smith, 2016) compared to observations from North Atlantic Hurricane database (HURDAT2; Landsea & Franklin, 2013). It was adopted here for both control and “future” simulations of Irene.

CPS describes the thermal evolutions of storms using three variables: the 900‐ to 600‐hPa geopotential thickness asymmetry (*B*), low‐level (900–600 hPa) thermal wind (*−V*_*T*_^*L*^), and upper‐level (600–300 hPa) thermal wind (*−V*_*T*_^*U*^). High values of *B* imply frontal systems. Positive thermal wind values indicate storms with warm core while negative thermal wind values indicate cold core systems. ET occurs when *B* exceeds 10 m (an empirical value based on a large set of storms) and completes when *−V*_*T*_^*L*^ drops below 0 (Evans & Hart, 2003). More details of CPS can be found in Evans and Hart (2003) and Hart (2003).

The CPS diagrams for both control and “future” simulations show similar thermal evolutions but with different thermal wind strengths (Figure S1). As Irene moved northward, it began to gain asymmetric characteristic following the increase of *B*. At the same time, the declining trend of 900‐ to 600‐hPa thermal wind indicated a decaying warm core structure. Compared to the control simulation, the storm in GW_ST generally shows a stronger warm core (Figure S1). In contrast, both GW and GW_AirT show a weaker warm core than the control simulation. More details on the warm‐core structure will be presented in later sections. Based on the CPS diagram analyses, we determined the ET onset and completion time (Table S2) and divided the life cycle of Irene into TC and ET phase. Consistent with Jung and Lackmann (2019), the onset of ET in “future” simulations is later than the control simulation. However, we do not find extended ET duration in “future” simulations compared to the control simulation.

### 2.3 Water Vapor Convergence Analyses

Water vapor convergence (CONV) is a key component of the moisture budget of TCs (e.g., Jiang et al., 2008; Wang et al., 2015). Given similar temporal change of water vapor convergence and storm rainfall rate (Figure S2), the water vapor convergence analyses provide the first‐order approximation of the storm rainfall rate. The water vapor convergence can be defined as

(1) $$\text{CONV}=\int_{\mathrm{bot}}^{\mathrm{top}}-\nabla \cdot \left(\mathrm{\rho qV}\right)dz\;$$

in which *CONV* is the water vapor convergence, *ρ* is the air density, *q* is the specific humidity, and *V* is the radial wind. The change of water vapor convergence can be written as

(2) $${\text{CONV}}_{\text{future}}-{\text{CONV}}_{\text{control}}=∆\left(\text{CONV}\right)=\int_{\mathrm{bot}}^{\mathrm{top}}-\nabla \cdot \left(\mathrm{\rho q}∆V+\rho ∆\mathrm{qV}+\rho ∆q∆V\right)dz\;$$

in which *CONV*_*future*_ and *CONV*_*control*_ on the left‐hand side indicate water vapor convergence from “future” and control simulation, respectively. The first and second terms on the right‐hand side indicate the contribution to changes of water vapor convergence from changes of radial wind and water vapor, respectively. The third term is nonlinear and approximately an order of magnitude smaller than the first two terms. The comparison of the first two terms is used to shed light on the relative contribution of dynamical and thermodynamic factors to storm rainfall rate changes.

## 3 Results

### 3.1 Responses of Storm Track and Intensity

**Figure 2:**
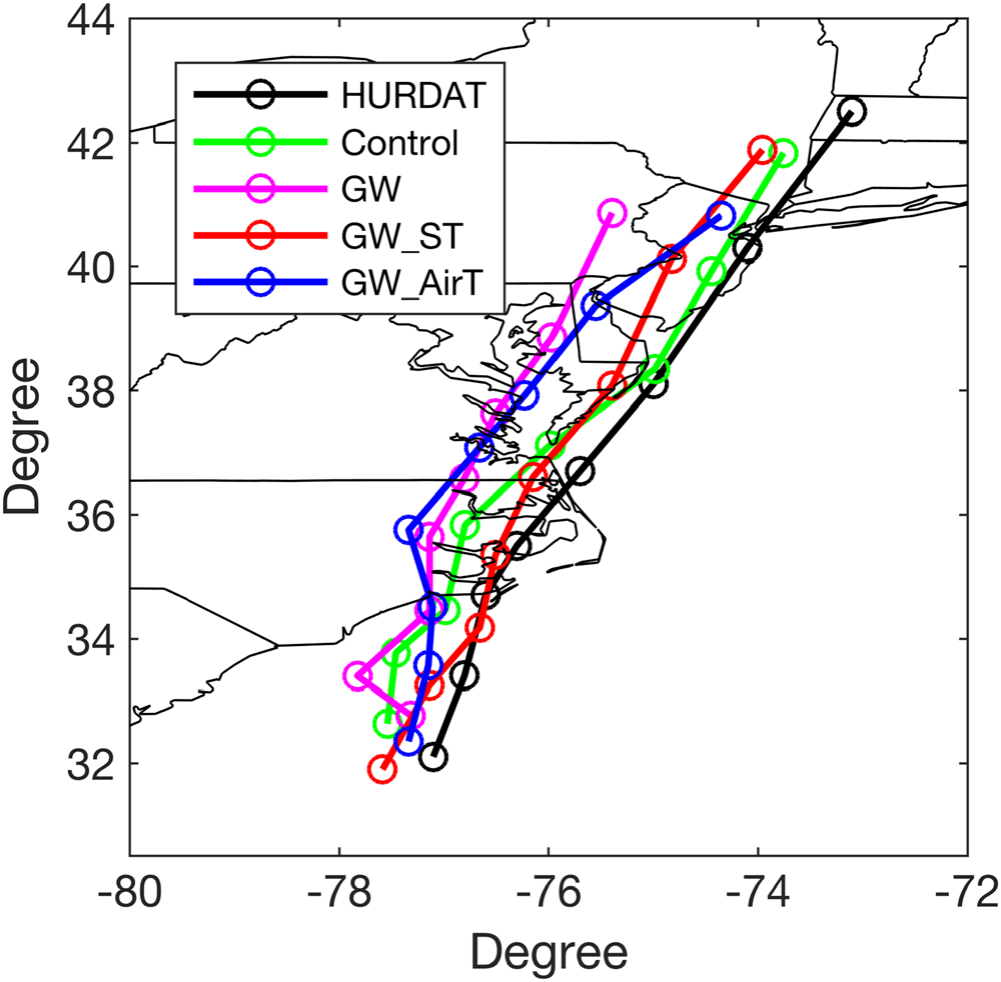
The storm track of Irene from HURDAT, control run, and three “pseudo‐global‐warming” simulations.

Before storm rainfall analyses, we first examined WRF‐simulated tracks (Figure 2). The simulated track of Irene from the control run and GW_ST shows a better match with HURDAT in comparison with GW and GW_AirT, suggesting that future thermodynamic changes (particularly tropospheric temperature) influence simulated tracks, possibly through dynamical effects induced by thermodynamic changes. Steering winds, calculated as the mean 1.5‐ to 10‐km wind (for both speed and direction) averaged over a 200‐ to 600‐km radius from the storm center, were used to understand track differences. The defined steering wind is a good indicator of storm motion because the two are largely oriented along the direction for the WRF simulations (Figure S3).

The storm simulated by the control run and GW_ST reaches higher latitudes than GW and GW_AirT, maybe due to their differences in meridional steering wind. For the period after 1800 UTC 27 August, the mean meridional steering wind for the control run (9.3 m/s) and GW_ST (10.3 m/s) are larger than GW (7.4 m/s) and GW_AirT (7.6 m/s). Compared to the control run, Irene from GW moves further inland (westward), consistent with the mean zonal steering wind (1.6 m/s for GW and 2.1 m/s for the control run). However, the consistency between zonal steering wind and Irene's track is not seen in GW_ST (1.6 m/s) and GW_AirT (1.9 m/s).

**Figure 3:**
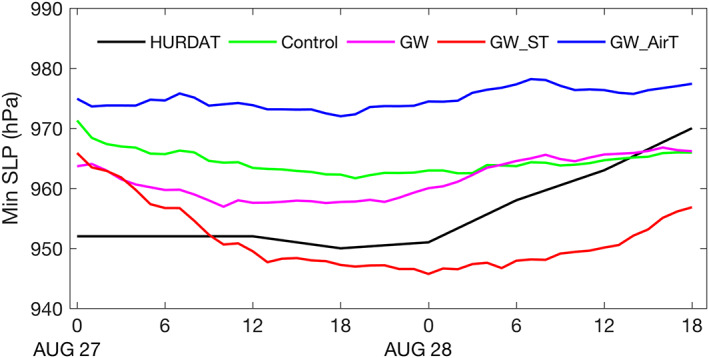
Minimum sea level pressure (hPa) from HURDAT, control run, and three “pseudo‐global‐warming” simulations.

Other factors (e.g., beta effect) should play a role in determining the track differences. In addition, zonal steering wind is less important than meridional steering wind due to a smaller magnitude. As shown in Figure 3, GW_ST attains deeper minimum sea level pressures than the control run, suggesting the effect of surface warming in storm intensification. In contrast, GW_AirT simulates lower intensity than the control run. The intensity simulated by GW lies between GW_ST and GW_AirT, due to the competing influence of surface and tropospheric warming.

### 3.2 Response of Storm Rainfall Rate and Structure

The WRF model shows good skill in simulating many key features of Hurricane Irene, including track, rainfall fields, and the ET process (see Liu & Smith, 2016, for details), providing the foundation to conduct numerical experiments for understanding Irene's response to future climate changes. The skin temperature over ocean and land surface shows contrasting changes under RCP8.5 scenario (Figure 1 a), probably linked to the limitations to evaporation over land (Byrne & O'Gorman, 2013; Sutton et al., 2007). Examination of the vertical profile of atmospheric temperature changes within the WRF domain shows enhanced upper tropospheric warming, the impact of which is discussed in later sections (Figure 1b). GW produces considerably higher storm rainfall for Irene, in particular, along coastal areas (Figures 1c and 1d). The changing patterns of rainfall fields for Irene are linked to changing properties of Irene's track (Figure 2). Storm center‐based analyses are used for the following results to address the influence of track shift.

**Figure 4:**
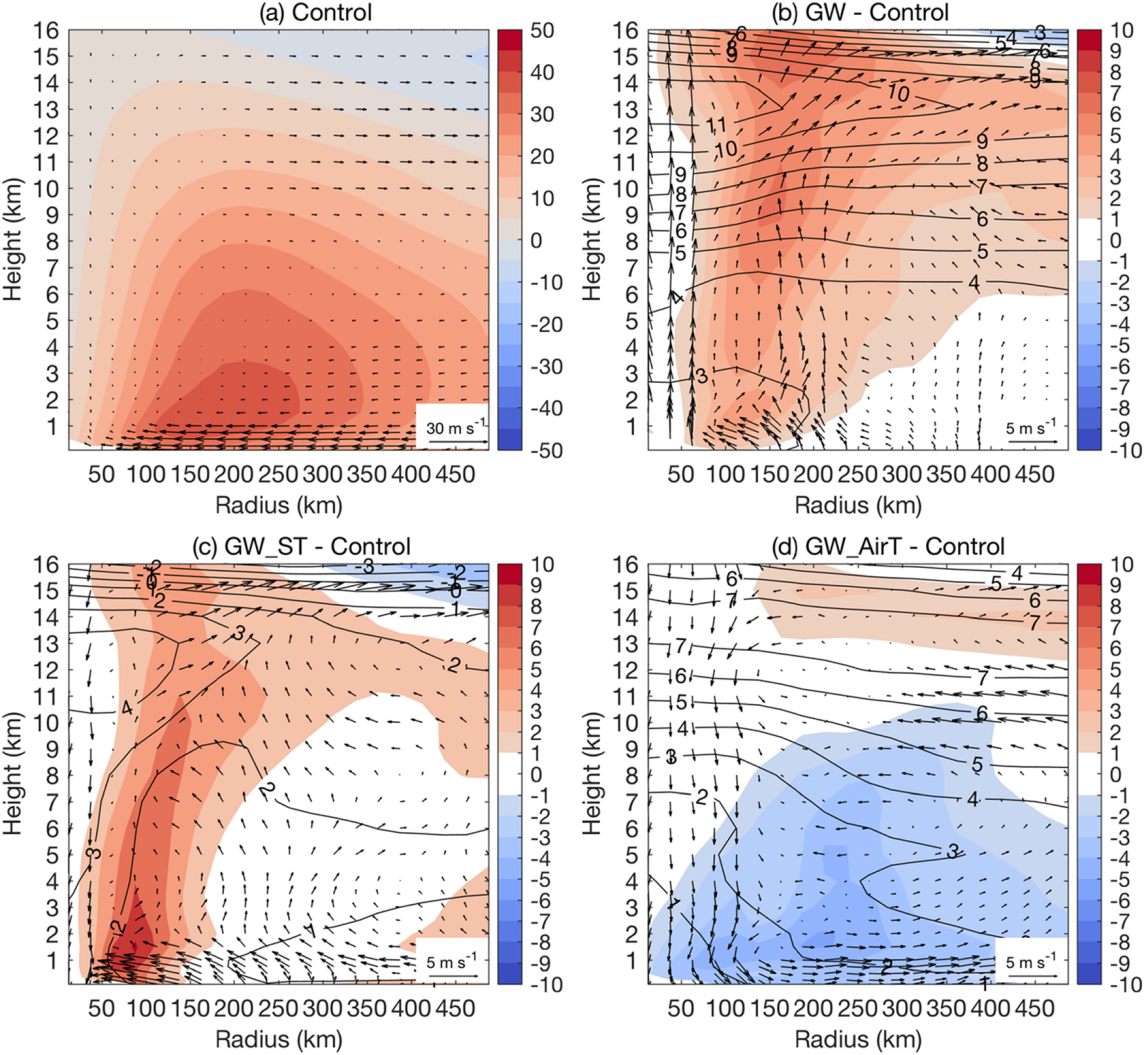
The radius‐height diagram of tangential wind (shaded; m/s), radial and vertical wind (arrows; m/s), and potential temperature (black contour; K) for (a) control simulation, and the difference between (b) GW, (c) GW_ST, and (d) GW_AirT simulation and the control simulation. For each simulation, the wind fields and potential temperature are averaged over both TC and ET phase. For illustration purpose the vertical wind has been multiplied by 50.

Table 1 shows the storm rainfall rate of Irene with respect to the storm phase and size for both control simulation and future projections. We first focus on the entire landfall period of Irene, including both TC and ET phases. The rainfall rate of Irene increases from 5.3 to 5.6 mm/hr as the averaging radius increases from 100 to 300 km, primarily as a result of strong outer rainbands during the ET phase (Atallah et al., 2007; Atallah & Bosart, 2003; Liu et al., 2017, 2018; Liu & Smith, 2016) and the weak nature of secondary circulation indicated by relatively small vertical updrafts in the inner core region (Figure 4 a).

**Table 1:** The Change of Storm Rainfall Rate of Irene With Respect to Phase and Radii

	Radius (km)	Control (mm)	GW (%)	GW_ST (%)	GW_AirT (%)
TC&ET	100	5.3	32	78	−12
	300	5.6	21	15	−12
	500	2.6	16	30	−15
TC	100	7.7	41	43	−16
	300	5.9	18	12	−12
	500	2.4	12	30	−18
ET	100	5.6	6	137	−25
	300	8.6	42	22	−2
	500 km	4.5	26	34	−6

*Note*. The rainfall change for the ET phase comes from the front quadrants of Irene.

The GW simulation produces higher storm rainfall rate than the control simulation, with the largest increase within the radius of 100 km (roughly the inner core region). We use the radius‐height diagram to examine underlying dynamical and thermodynamic properties of Irene responsible for rainfall rate changes. Relative to the control simulation, GW‐projected Irene shows increased low‐level radial inflow and enhanced upper‐level outflow up to 15 km, which in combination with increased updrafts in inner core regions forms an enhanced deep secondary circulation (Figure 4b). The enhancement of secondary circulation, broadly consistent with the increased storm intensity in GW (Figure 3), highlights the role of dynamical factors in the inner core rainfall rate increase.

Echoing the radius‐height diagram analyses, CONV analyses further highlight the importance of strengthened secondary circulation in enhancing the inner core storm rainfall, although the contribution to CONV (13%) is slightly lower than that from atmospheric moistening (18%) (Table 2). Along with the increase of averaging radius, GW produces a lower increase of storm rainfall rate. For a radius of 500 km, the rainfall rate increases by 16% (Table 2), close to the rate expected by the Clausius‐Clapeyron relation for a lower tropospheric temperature increase of approximately 3 K (Figure 4b). The CONV analyses echo this point by showing slight negative contribution from dynamical factors (−4%). In summary, vortex strengthening plays an important role in enhancing storm rainfall rate in the inner bands through enhanced secondary circulation, which gradually diminishes as one moves to outer rainbands (e.g., Knutson et al., 2013).

**Table 2:** Attribution of Water Vapor Convergence for Each Perturbation Experiment (Rows) to Wind Fields, Atmospheric Water Vapor Contents and the Nonlinear Term, (Last Three Columns, Respectively)

		Δ*V* (%)	Δ*q* (%)	Δ*V* and Δ*q* (%)
GW	100 km	13	18	1
	500 km	−4	24	−1
GW_ST	100 km	59	6	2
	500 km	16	1	1
GW_AirT	100 km	−18	13	−3
	500 km	−22	20	−4

### 3.3 Roles of Surface Temperature and Air Temperature

Results from the GW projection are used along with GW_ST and GW_AirT to shed light on the role of surface warming and atmospheric warming in the future change of Irene. In response to CO_2_ forcing there is enhanced upper tropospheric warming relative to the surface (Figure 1b), which would lead to a more stable troposphere that is less conducive to TC development (e.g., Hill & Lackmann, 2011; Shen et al., 2000). In GW_AirT, the vertical profile of potential temperature shows increased warming with height (Figure 4d), implying enhanced stabilization of tropospheric lapse rate in the local storm environment. For Irene, GW_AirT simulates lower storm rainfall rates than the control simulation for all averaging radii (Table 1), consistent with the weakened secondary circulation seen in the radius‐height diagram by the decrease of low‐level inflow, upper‐level outflow, and inner core updrafts (Figure 4d).

The weakening of Irene in GW_AirT is also indicated by the decrease of tangential wind (Figure 4d) and storm intensity (Figure 3). Further, the CONV analyses show that, although the enhanced water vapor content due to atmospheric warming tends to increase rainfall rate, the reduced strength of inflow plays a more important role, which results in a decrease of storm rainfall rate (Table 2). Relative to GW_AirT, the offsetting effect of lapse rate stabilization in storm intensity and rainfall rate seems to be enhanced in GW as seen in the greater increase of upper tropospheric warming, primarily as a result of larger latent heating releases (Figures 4b and 4d).

The isolated influence of surface temperature change on future storm activity was explored with GW_ST. For the radius of 100 km, the increase of storm rainfall rate in GW_ST (78%) is nearly twice that in GW (32%) (Table 1). This contrast of rainfall rate increase is dominated by the ET phase of Irene, detailed analyses of which are provided later.

Attribution analyses of CONV changes are examined to identify sources of storm rainfall rate changes. The enhanced radial inflow plays a dominant role in the future change of 100‐km water vapor convergence for GW_ST, while a relatively modest role for GW (Table 2). The contrast of the two simulations echoes the fact that GW_ST produces a larger increase of storm intensity than GW (Figure 3) and reflects the role of enhanced upper tropospheric warming (the enhanced stabilization of tropospheric lapse rate in GW is not seen in GW_ST; Figures 4b and 4d) in offsetting the strengthening of secondary circulation. This is consistent with the lower storm thermodynamic efficiency in GW due to much warmer outflows (Emanuel, 1986; Hill & Lackmann, 2011).

In contrast to enhanced radial inflow, atmospheric moistening plays a marginal role in the increase of water vapor convergence in GW_ST, much lower than in GW (Table 1). This contrast of GW_ST and GW scenarios highlights the future change of specific humidity in terms of the height‐radius diagram. GW simulates a considerable increase of specific humidity, particularly in the lower troposphere (Figure S4a), while GW_ST simulates only a marginal increase of water vapor content (Figure S4b).

### 3.4 Contrasting Response at TC and ET Phases

Analyses of storm‐direction‐oriented rainfall rates based on the control simulation highlight the contrast of rainfall fields between TC and ET phase (Figure S5). The rainfall fields during the ET phase, compared to the TC phase, show less symmetric characteristics and extend to larger areas further from the storm center (Figure S5; see Liu and Smith (2016) for more details). Thus, we examined Irene's response to climate warming for the two phases, respectively, the comparison of which was expected to advance the understanding of storm rainfall changes and underlying physical mechanisms.

**Figure 5:**
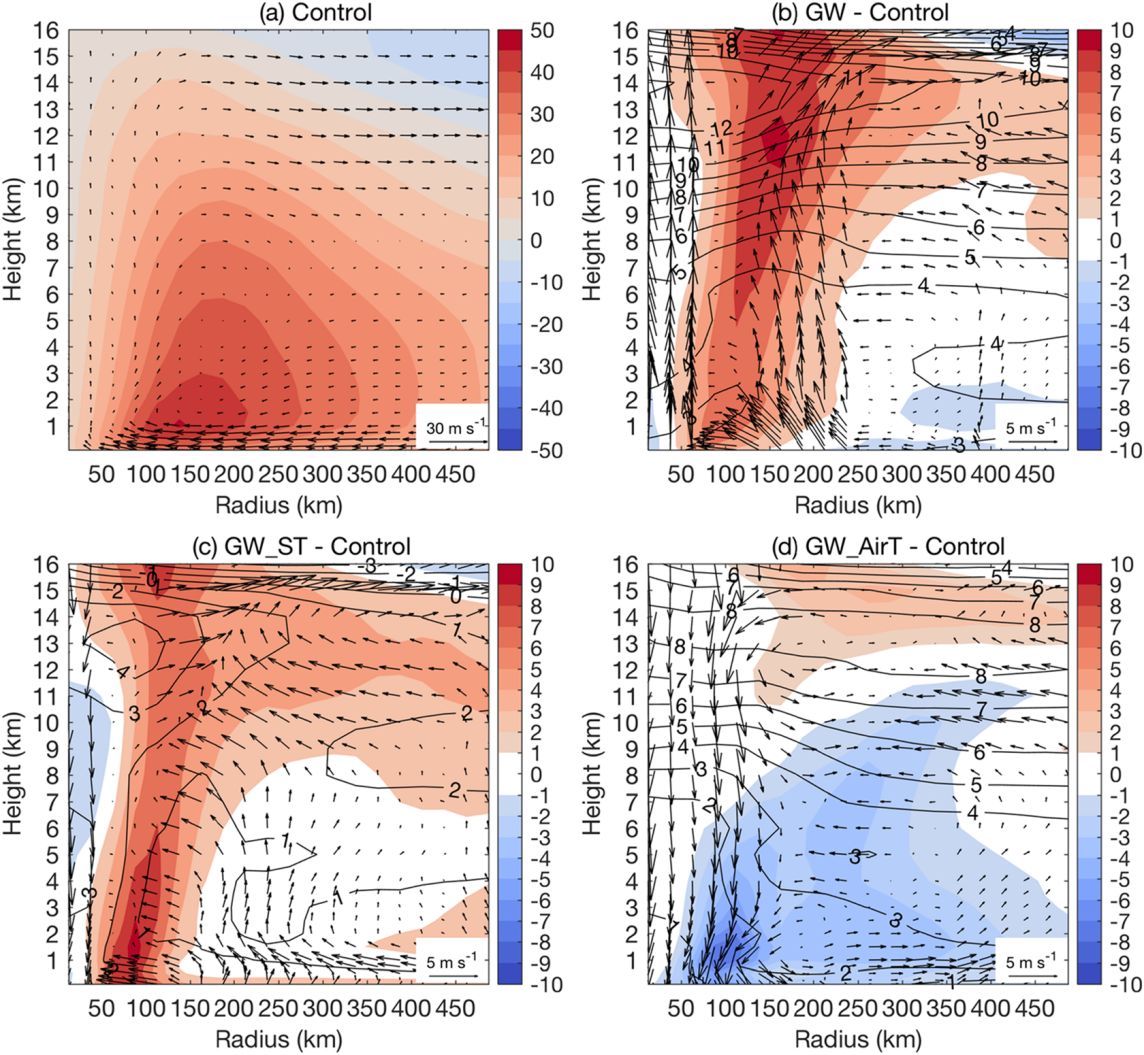
As in Figure 4 but for the TC phase only.

For the TC phase (Figure 5a), the radial and vertical wind fields in the radius‐height diagram suggest a slightly stronger secondary circulation than the entire landfall period that includes both TC and ET phases (TC&ET hereafter for convenience; Figure 4a). As seen in the reduced radius of maximum wind (Figures 4a and 5a), Irene at the TC phase has a tightened warm core, consistent with the higher 100‐km storm rainfall rate (7.7 mm/hr) relative to the TC&ET phases (5.3 mm/hr). These differences can be traced to the decaying warm core during the ET phase, more discussion of which will be provided later. GW_AirT for the TC phase, similar to the TC&ET phases, simulates reduced rainfall rates for an averaging radius ranging from 100 to 500 km (Table 1), consistent with the decreased strength of secondary circulation (Figure 5d) and storm intensity (Figure 3).

GW produces a higher 100‐km rainfall rate increase for the TC phase (41%) than the TC&ET phases (32%) (Table 1), in accordance with greater strengthening of secondary circulation (Figures 4b and 5b). Although GW_ST produces a comparable 100‐km rainfall rate increase to the GW run (Table 1), there are noticeable differences between the two in terms of storm structures, for example, the vertical distribution of tangential wind increase. The increase of tangential wind in GW_ST reaches a maximum at relatively low levels and then gradually declines with height. The maximum increase of tangential wind in GW, on the contrary, occurs at the 12‐km level and shows a decreasing trend to the ground. This is consistent with the fact that GW_ST has a higher warm core strength than GW in terms of thermal wind (Figure S1). The warmer upper‐level air temperature in GW would lead to the lifting of freezing level that favors the efficiency of uplift in the upper level, which may lead to more upper‐level latent heating than other simulations. To balance the increased pressure gradient due to enhanced latent heating, GW produces a larger increase of upper‐level tangential wind.

For ET phase analyses, we focus on the front quadrants in which Irene rainfall is concentrated. Starting from the control simulation, the storm rainfall rate grows higher as the averaging radius increases from 100 to 300 km. The spatial expansion of rainfall fields is a distinct feature of ET phase (e.g., Atallah & Bosart, 2003; Colle, 2003; Liu et al., 2017; Liu et al., 2018; Liu & Smith, 2016) from TC phase in which the storm rainfall rate generally declines with increased averaging radius.

**Figure 6:**
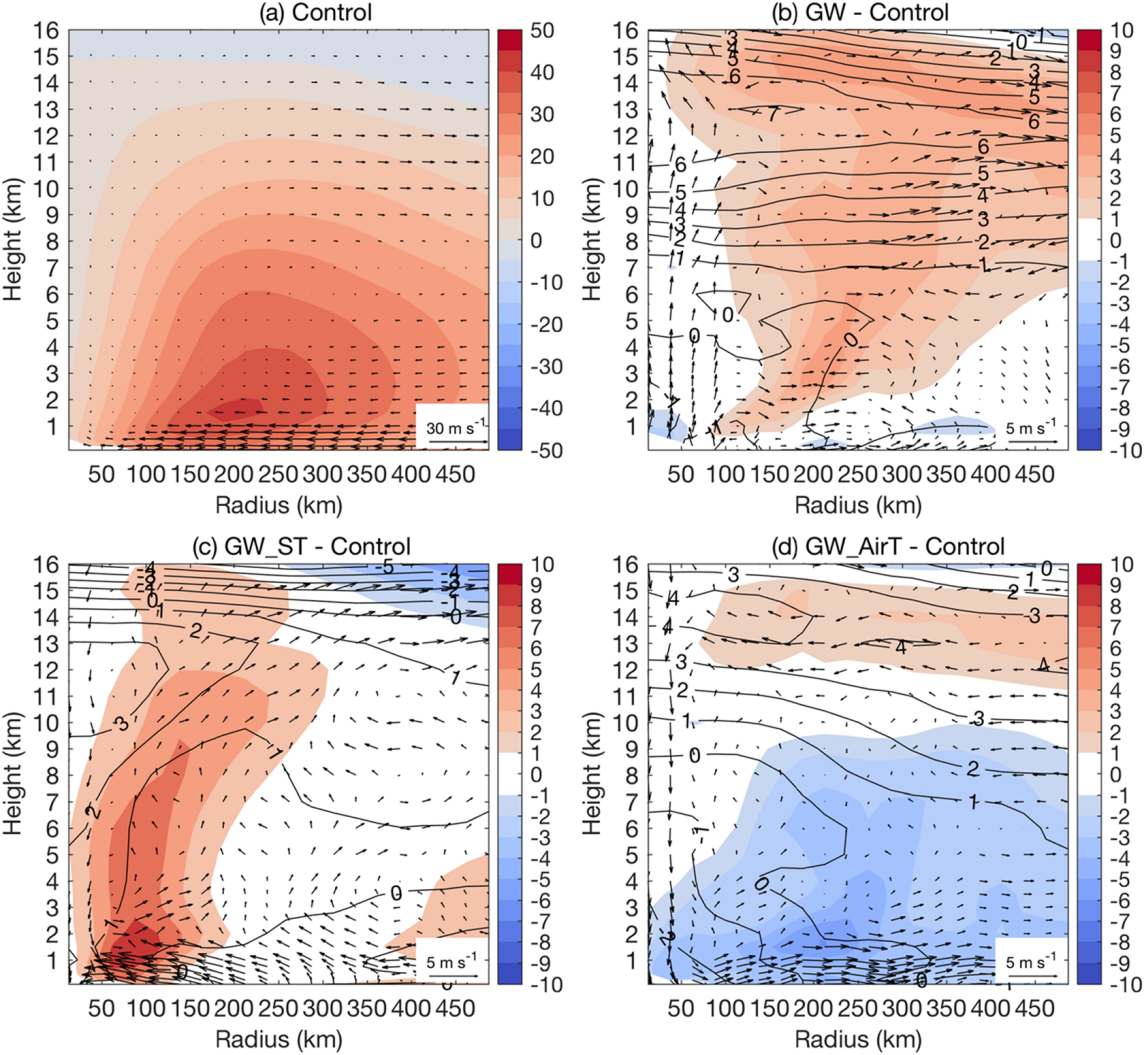
As in Figure 4 but for the ET phase only.

The contrast of the two phases is also reflected in the radius‐height diagram in which Irene at the ET phase shows a weaker warm core relative to the TC phase, as indicated by the lower low‐level tangential wind and larger radius of maximum wind (Figures 5a and 6a). Jung and Lackmann (2019) found decreased storm rainfall rates in the outer rainbands of Irene at the ET phase as climate warms, which was attributed to the east shift of storm track for the future Irene. In this study, Irene's track in the warmer climate shifted to the west of that in the present climate (Figure 2). To mitigate the impact from the shift of storm tracks, Jung and Lackmann (2019) carried out partially idealized simulations in which the land and orography are removed. The present results are consistent with those of Jung and Lackmann (2019) in showing large increases of future storm rainfall rate during the ET phase.

For the ET phase, GW_ST produces a striking increase of (137%) of storm rainfall rate for an averaging radius of 100 km, much higher than GW (6%) (Table 1). This difference between GW_ST and GW at the ET phase (137% vs. 6%), compared to the TC phase (43% vs. 41%), accounts for the major part of the contrasting inner core rainfall rate increase over the landfall period (78% vs. 32%). This difference also echoes the simulated change of radius‐height diagram.

Relative to the control simulation, GW_ST simulates enhanced tangential winds throughout the troposphere with the maximum increase in the inner core region (within 150 km from the storm center) and a strengthening of secondary circulation (Figure 6c) during the ET phase, implying the maintenance of inner core convective processes that decay in the control simulation. This supports the striking increase of storm rainfall rate (137%) within the radius of 100 km. In contrast to GW_ST, GW does not see enhanced tangential winds within 100 km from the storm center at the ET phase (Figure 6b), supporting the marginal increase of storm rainfall rate (6%). The enhancement of tangential wind in GW covers a large area (100–450 km of the storm center) with the maximum at a radius of around 250 km (Figure 6b). This is consistent with the relatively large increase of storm rainfall rate (42%) at an averaging radius of 300 km, larger than the rate expected from the Clausius‐Clapeyron relation. The enhanced outflow at heights of 7–14 km also suggests the important role of dynamical processes in the rainfall rate increase (Figure 6b). GW_AirT, opposite to GW_ST and GW, produces reduced storm rainfall rates for various averaging radii at the ET phase (Table 1), consistent with the weakened strength of circulation (Figure 6d).

## 4 Summary

Landfalling TCs are one of the principal agents of extreme rainfall and flooding in the eastern United States (e.g., Villarini et al., 2011; Villarini, Goska, et al., 2014), with ET playing an important role in rainfall enhancement as illustrated in various storm events (e.g., Atallah & Bosart, 2003; Colle, 2003; DiMego & Bosart, 1982; Liu & Smith, 2016). Studies examining the response of landfalling storms to anthropogenic forcings are expected to improve our understanding of future TC‐related flood risks. However, compared to TCs, there are fewer studies focusing on the response of transitioning storms to climate change (Baatsen et al., 2015; Haarsma et al., 2013; Jung & Lackmann, 2019; Liu et al., 2017, 2018). Hurricane Irene (2011) is among the TC storm events that produced the most extreme flooding in recent record with ET as a key element responsible for extreme rainfall (Liu & Smith, 2016), providing an ideal case to examine the influence of GW on transitioning storms (Liu et al., 2018).

We conducted numerical simulations of Irene, including a control simulation and “pseudo‐global‐warming” projections with the WRF model. The initial and boundary conditions for the “pseudo‐global‐warming” projections were produced by adding to historical input data the thermodynamic components of large‐scale climate changes from simulations of the CESM model in response to the RCP8.5 (“business as usual”) 21st century scenario. A close examination of responses of Irene for both TC and ET phases to greenhouse gas emissions was conducted to improve our understanding of the change of storm rainfall and the underlying physical mechanisms. Major findings of the paper are summarized as follows.

1. The GW simulation using CESM‐projected surface and atmospheric temperature under RCP8.5 scenario produces increased storm rainfall rates over the control simulation for various averaging radii. The rainfall rate shows the largest increase (32%) in the inner core regions (roughly 100 km from the storm center), which decreases with respect to the averaging radius, consistent with that the significant increase of tangential wind and low‐level radial inflow in the inner core is not seen in outer bands. Without future surface temperature, GW_AirT produces decreased storm rainfall rates and weaker circulation strengths relative to the control simulation, primarily as a result of the stabilization of tropospheric lapse rate due to enhanced upper‐level warming. Without the constraint of future air temperature, GW_ST simulates a larger increase of storm rainfall rate and secondary circulation strength than GW in the inner core regions.
2. Water vapor convergence, a key feature of TC rainfall production, provides a path for quantitative understanding of factors influencing storm rainfall rates through factor decomposition analyses. In GW, atmospheric moistening and enhanced radial inflow are comparably important to the rainfall rate increase in the inner core regions. But for the rainfall rate change over an averaging radius of 500 km, the increased atmospheric water vapor content becomes a dominant factor. The rainfall rate increase in GW_ST, unlike GW, is dominated by the enhanced radial inflow. For GW_AirT, the change of rainfall rate is the result of competition between increased water vapor and decreased inflow strength. Water vapor convergence analyses highlight the important role of dynamical processes in rainfall rate changes.
3. The change of storm rainfall rate shows contrasting responses to anthropogenic GW between TC and ET phases. In the GW projection of Irene, the TC phase shows a significant increase of storm rainfall rate in the inner core region, which shifts to outer rainbands as Irene undergoes ET, consistent with the location of maximum tangential wind increase and the change of secondary circulation strength. In GW_ST, Irene shows a much larger increase of inner core rainfall rate for the ET phase than the TC phase, primarily due to delayed decaying of convective processes in the inner core region relative to the control simulation. The contrasts between GW and GW_ST highlight the role of atmospheric temperature change in storms' response to anthropogenic forcings.

In summary, the surface warming (see GW_ST) is expected to intensify TCs while the enhanced stabilization of the tropospheric lapse rate (see GW_AirT; Hill & Lackmann, 2011) weakens TCs, indicating an offsetting effect (Knutson & Tuleya, 2004; Shen et al., 2000). However, as seen in various global climate models, the two are positively correlated; that is, model projections with greater surface warming tend to have greater maximum tropospheric warming aloft (Hill & Lackmann, 2011). In addition, the upper tropospheric warming, compared to surface warming, shows a larger spread among global climate model projections (Hill & Lackmann, 2011). These factors highlight the sources of uncertainty for the influence of environmental thermodynamic changes on storms. For improved understanding of future storm risks, future work should extend these results to include more global climate models and more storm cases to quantify related sources of uncertainty.

## Supporting information

Supporting Information S1
