# Supplementary material for: Response of Extreme Rainfall for Landfalling Tropical Cyclones Undergoing Extratropical Transition to Projected Climate Change: Hurricane Irene (2011)
Source: Earths Future. 2020 Mar 3;8(3):e2019EF001360. doi: 10.1029/2019EF001360 (PMC7375049; doi:10.1029/2019EF001360)
Supplement: Supplementary file 1 — Supporting Information S1 [file EFT2-8-e2019EF001360-s001.pdf]

Earth's Future

Supporting Information for

Response of extreme rainfall for landfalling tropical cyclone undergoing extratropical transition to projected climate change: Hurricane Irene (2011)

M.F. Liu[1], L. Yang[2,1], J. A. Smith[1], G. A. Vecchi[3,4]

[1]Department of Civil and Environmental Engineering, Princeton University, Princeton, New Jersey, USA

[2]School of Geography and Oceanic Science, Nanjing University, Nanjing, Jiangsu Province, China

[3]Department of Geosciences, Princeton University, Princeton, New Jersey, USA

[4]Princeton Environmental Institute, Princeton University, Princeton, New Jersey, USA

Contents of this file

Figures S1 to S6
Tables S1 to S2

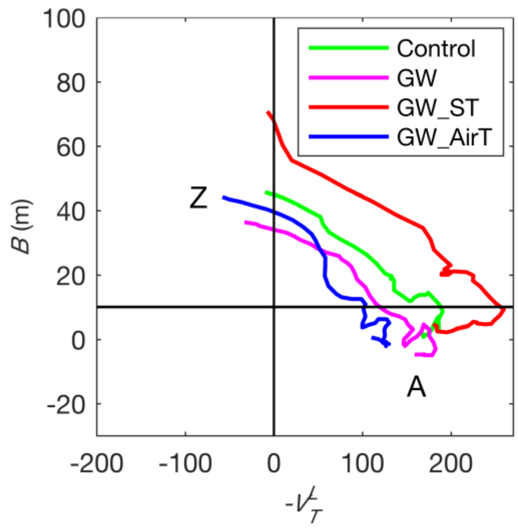

Figure S1. The cyclone phase space diagram with 900-600-hPha thermal wind (-V_{TL}) versus 900-600-hPa thickness asymmetry (*B*; m) for the control simulation and three 'pseudo-global-warming' simulations. 'A' indicates the storm initiation while 'Z' indicates the end of the simulation. The horizontal black line is the threshold of *B* (10 m). Upper quadrant of the line indicates asymmetric or frontal system while lower quadrant indicates symmetric system. The vertical black line is the threshold of -V_{TL} (0). Left quadrant of the line indicates cold core while right quadrant indicates warm core.

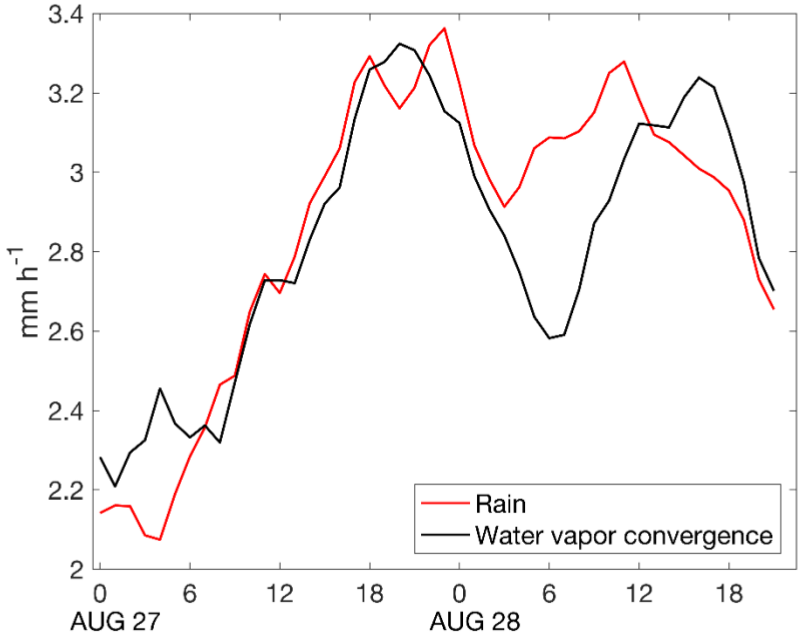

Figure S2. The time series of storm rainfall rate averaged within 500 km from the storm center and corresponding water vapor convergence from the control simulation of Irene.

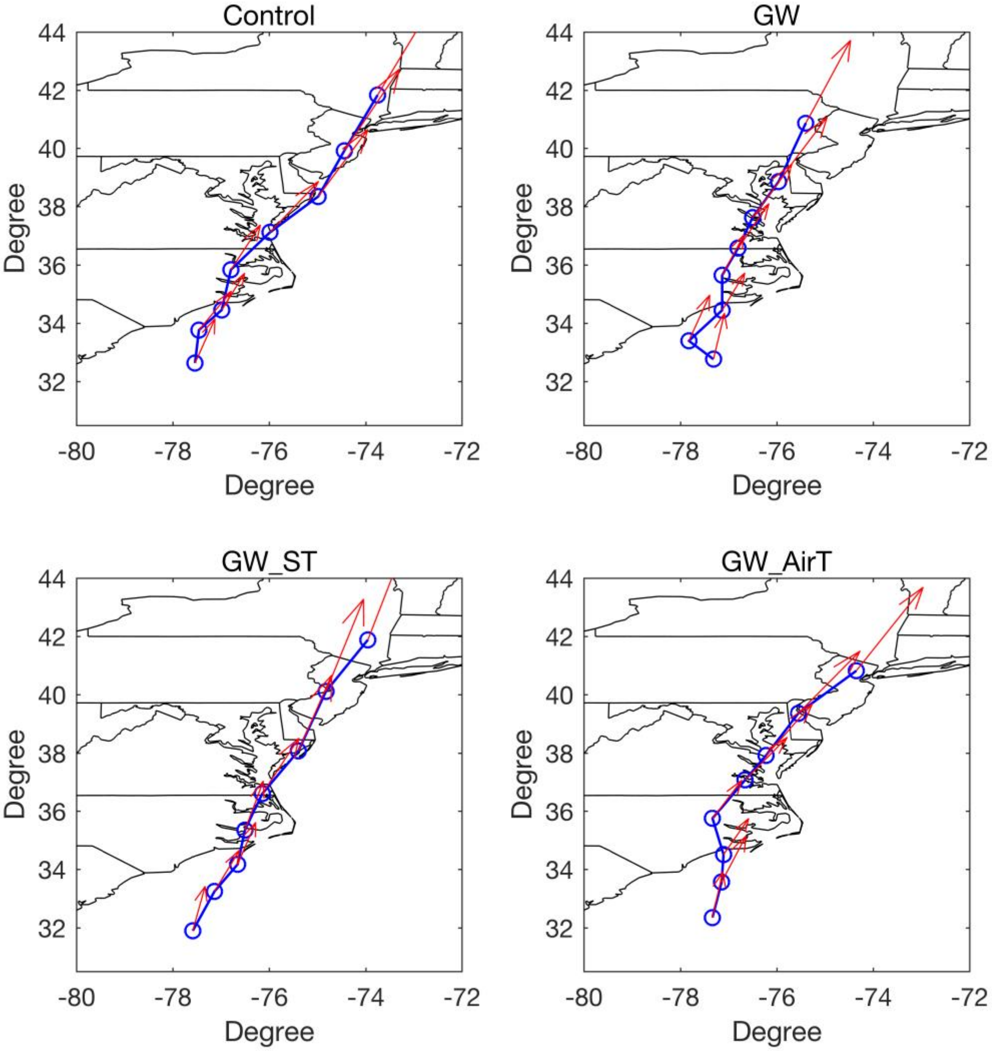

Figure S3. Simulated storm tracks (blue lines and dots) and steering wind (red arrow) of Irene from control run and three 'pseudo-global-warming' simulations.

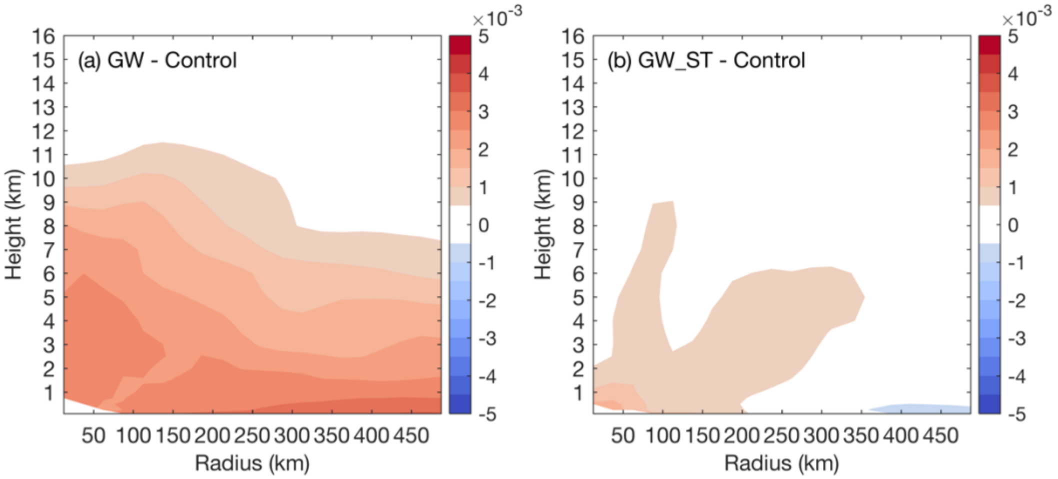

Figure S4. The radius-height diagram of the difference of specific humidity (kg kg-1) between (a) GW, (b) GW_ST and the control simulation. The specific humidity is averaged over both TC and ET phase.

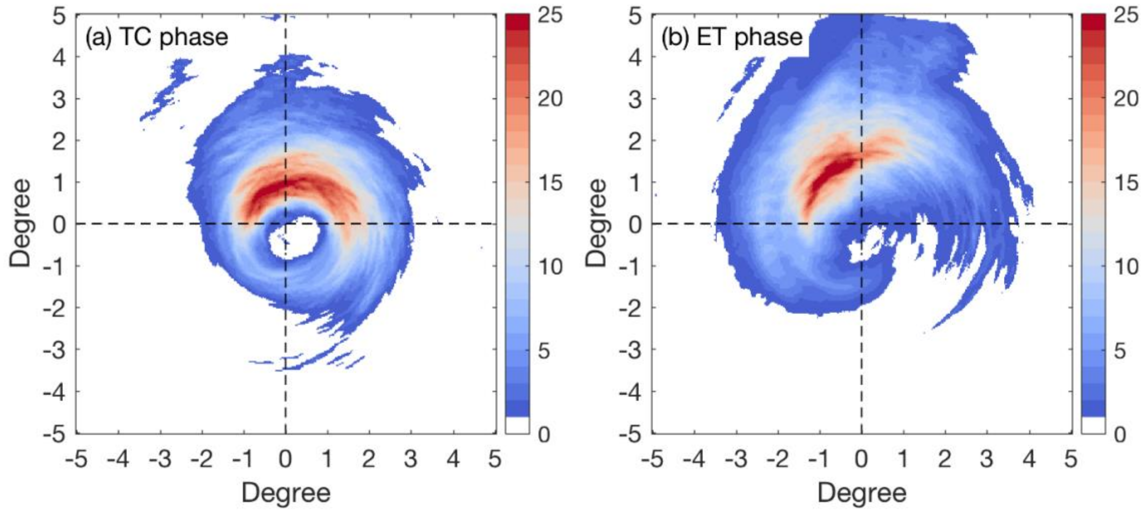

Figure S5. The storm-direction-oriented rainfall rates (mm h^{-1}) for (a) TC phase and (b) ET phase.

Domain	D1	D2
Dimensions (*x, y*)	340 × 422	730 × 940
Grid size (km)	9	3
Output interval (h)	3	1
Cumulus Scheme	Kain-Fritsch scheme	None
Microphysics Scheme	WSM6	WSM6
Vertical layers	28	28
Longwave radiation	RRTM scheme	RRTM scheme
Shortwave radiation	Dudhia scheme	Dudhia scheme
Surface layer	Monin-Obukhov Scheme	Monin-Obukhov Scheme
Land surface	Noah land surface model	Noah land surface model
Boundary layer	YSU	YSU
Land use	MODIS 2 m	MODIS 30 s

Table S1. Overview of WRF physical options.

WRF runs	ET onset time	ET completion time
Control	1800UTC27	0400UTC29
GW	0400UTC28	0300UTC29
GW_AirT	0200UTC28	0100UTC29
GW_ST	1900UTC27	0400UTC29

Table S2. The onset and completion time of ET for all WRF runs.
